# Acknowledgment of Invited Editors

**DOI:** 10.1128/mBio.00199-16

**Published:** 2016-03-08

**Authors:** Arturo Casadevall

## EDITORIAL

On behalf of the Editors of *mBio*, I gratefully acknowledge the following individuals, who served as Invited Editors for the journal in 2015. While not members of the Board of Editors, Invited Editors serve an important role in the review process. Invited Editors are experts in their fields of research who add an additional level of quality to the review process. An Editor may assign a paper to an Invited Editor when he/she would like to have an additional expert opinion of the reviews or when the subject area falls outside the Editor’s primary area of expertise.

The time and effort expended by the following experts in handling articles have been essential to ensuring the high quality of our publications, and their help is greatly appreciated.

Eric Alm

J. Andrew Alspaugh

Lisa Alvarez-Cohen

Rama Rao Amara

David Andes

Gustavo Arrizabalaga

Jimmy D. Ballard

James Bann

Joseph T. Barbieri

Dennis A. Bazylinski

Thomas Bernhardt

Melanie Blokesch

Elhanan Borenstein

Jon P. Boyle

Roland Brosch

Harlan D. Caldwell

Andrew Camilli

Emmanuelle Charpentier

Carton W. Chen

Jose C. Clemente

Fevzi Daldal

Frank van de Veerdonk

Willem M. de Vos

Thomas Dick

Dirk P. Dittmer

Tamara L. Doering

Paul Dunman

Gary M. Dunny

Roselyn J. Eisenberg

Sean Elliott

Francis Ennis

Jose A. Este

Edward J. Feil

Reinhard Fischer

Vance G. Fowler

Dara W. Frank

James A. Fraser

Philippe Gerard

Gustavo H. Goldman

Andrew Goodman

Heidi Goodrich-Blair

Bill Hanage

Simon R. Harris

Anton Hartmann

Xiaosong He

David R. Hendrixson

Joseph M. Hyser

Ivan Junier

Sheryl Justice

Joerg Kaemper

Fatah Kashanchi

Daniel B. Kearns

Damian J. Krysan

Michael T. Laub

Wayne Lencer

Bruce R. Levin

Petra Anne Levin

Susan Liebman

Xiaorong Lin

Peter N. Lipke

Victor de Lorenzo

Stephen Lory

Ana Maldonado

Anthony W. Maresso

Anthony T. Maurelli

Houra Merrikh

Samuel I. Miller

Cesare Montecucco

Donald A. Morrison

Samuel M. Moskowitz

Tarek Msadek

Mihai G. Netea

Mark Nitz

George O'Toole

R. Thane Papke

Eleftherios T. Papoutsakis

John S. Parker

Matthew R. Parsek

Vicente Planelles

Laurent Poirel

Maria I. Pozo

Rajendra Prasad

Venigalla Rao

David A. Rasko

Federico Rey

Eleanor M. Riley

Jason W. Rosch

Erica Ollmann Saphire

Thomas Mitchell Schmidt

Thamarai Schneiders

Julia A. Segre

Angela Sessitsch

Anita Sil

Eric P. Skaar

David Skurnik

Thomas E. Smithgall

Alfred Michael Spormann

Christina L. Stallings

Boris Striepen

Curtis Suttle

John M. Taylor

Teresa Thiel

Justin Adam Thornton

Jan Tommassen

Bruce E. Torbett

Victor J. Torres

Mark Travassos

Ana Traven

Elaine I. Tuomanen

Gilles P. van Wezel

Fabrice Vavre

Alan W. Walker

Gary P. Wang

David Wang

Roy D. Welch

Marvin Whiteley

Kelly Wrighton

Otto O. Yang

